# Genome Sequencing of a Camelpox Vaccine Reveals Close Similarity to Modified *Vaccinia virus* Ankara (MVA)

**DOI:** 10.3390/v12080786

**Published:** 2020-07-23

**Authors:** Maurilia Marcacci, Abdelmalik I. Khalafalla, Zulaikha M. Al Hammadi, Federica Monaco, Cesare Cammà, Mohammed F. Yusof, Saeed M. Al Yammahi, Iolanda Mangone, Fabrizia Valleriani, Mohamed A. Alhosani, Nicola Decaro, Alessio Lorusso, Salama S. Almuhairi, Giovanni Savini

**Affiliations:** 1 Istituto Zooprofilattico Sperimentale dell’Abruzzo e Molise, 64100 Teramo, Italy; f.monaco@izs.it (F.M.); c.camma@izs.it (C.C.); i.mangone@izs.it (I.M.); f.valleriani@izs.it (F.V.); a.lorusso@izs.it (A.L.); g.savini@izs.it (G.S.); 2 Department of Veterinary Medicine, University of Bari, Valenzano, 70010 Bari, Italy; nicola.decaro@uniba.it; 3 Veterinary Laboratories Division, Abu Dhabi Agriculture and Food Safety Authority (ADAFSA), Abu Dhabi 52150, UAE; abdelmalik.khalafalla@ADAFSA.GOV.AE (A.I.K.); zulaikha.alhammadi@ADAFSA.GOV.AE (Z.M.A.H.); Mohd.Yusof@ADAFSA.GOV.AE (M.F.Y.); saeed.alyammahi@ADAFSA.GOV.AE (S.M.A.Y.); mohamed.a.alhosani@ADAFSA.GOV.AE (M.A.A.); salama.almuhairi@ADAFSA.GOV.AE (S.S.A.)

**Keywords:** camelpox, vaccine, *Vaccinia virus*, next-generation sequencing

## Abstract

Camelpox is a viral contagious disease of Old-World camelids sustained by *Camelpox virus* (CMLV). The disease is characterized by mild, local skin or severe systemic infections and may have a major economic impact due to significant losses in terms of morbidity and mortality, weight loss, and low milk yield. Prevention of camelpox is performed by vaccination. In this study, we investigated the composition of a CMLV-based, live-attenuated commercial vaccine using next-generation sequencing (NGS) technology. The results of this analysis revealed genomic sequences of Modified *Vaccinia virus* Ankara (MVA).

## 1. Introduction

Camelpox is a viral contagious disease of Old-World camelids and is characterized by mild, local skin or severe systemic infections. This disease may have a major economic impact due to significant losses in terms of morbidity and mortality, weight loss, and low milk yield [1]. The causative agent is *Camelpox virus* (CMLV), a double-stranded (ds) DNA virus belonging to the genus *Orthopoxvirus* (OPXV) of the family *Poxviridae*, subfamily *Chordopoxvirinae*. Twelve species are recognized within this genus, and the most notable include *Variola major virus* (VARV), *Vaccinia virus* (VACV), and *Cowpox virus* (CPXV). The *Orthopoxvirus* genome is composed of several structures. Two inverted terminal repeats (ITRs), identical in sequence, are present at the ends of the dsDNA genome and form covalently closed hairpin structures [2]. In addition, ITRs consist of further sequence elements, including repeat and coding regions. Furthermore, left and right terminal regions flank a central conserved region. Left and right terminal regions contain open reading frames (ORFs) encoding for proteins involved in host interactions (host range, immunomodulation, and pathogenicity), while the central region encloses ORFs encoding for proteins involved in essential viral replicative processes [3]. However, differences in the genomes could be observed between the viral species of this genus. First, genome size may vary. Taking into account VACV, CPXV, and CMLV, it ranges from 194,000 (VACV) to 229,000 (CPXV). Accordingly, genome constellations are also variable. As an example, genomic differences between CMLV and other OPXVs are greater in terminal regions due to small and large nucleotide (nt) insertions, deletions, and translocations [4]. Such deletions may also be due to cell-culture adaption. For instance, Modified *Vaccinia virus* Ankara (MVA), used during the last years of the WHO smallpox eradication campaign, shows a significantly smaller genome compared to extant VACV strains likely as a consequence of the massive passaging (over 570 times) in chicken embryo fibroblasts [5]. While other OPXVs can infect various hosts, CMLV seems to be restricted to a single host, and camels usually get infected by contact with infected animals through skin abrasions or via aerosol [1]. The virus can also spread in the environment either in water or on fomites [6,7]. Unlike other OPXVs, whose genome sequences are highly represented in public databases, the number of CMLV sequences is still very limited. Prevention and control of camelpox can be achieved using live-attenuated and inactivated vaccines. Recently, it was discovered that an attenuated CMLV vaccine, prepared and commercialized in the Kingdom of Saudi Arabia (KSA) using strain Jouf-78, contained VACV rather than CMLV [8]. In this study, by employing modern NGS technology, we attempted to determine and analyze the whole genome sequence (WGS) of another commercially available CMLV vaccine, the Ducapox vaccine.

## 2. Materials and Methods

### 2.1. Ducapox Vaccine

The live-attenuated Ducapox vaccine (batch number DPV0816, manufacture date August 2016, expiration date August 2017) contains 10^6.5^ TCID_50_/mL of strain CaPV298-2, which was isolated in the United Arab Emirates (UAE) and attenuated by 120 serial passages into Vero cells [9]. The vaccine, originally developed by Onderstepoort Biological Products [10,11] and produced by Highveld Biologicals, Republic of South Africa (RSA), is currently manufactured by the Central Veterinary Research Laboratory (CVRL, Dubai, UAE). A total volume of 11 mL of sterile, distilled water has been used for the reconstitution.

### 2.2. NextSeq500 (Illumina) Sequencing

DNA purification was performed starting from 200 µL of reconstituted sample using the High Pure Viral Nucleic Acid Kit (Roche, Basel CH, Switzerland) according to the manufacturer’s guidelines. DNA was then quantified by Qubit dsDNA HS assay (Thermo Fisher Scientific, Waltham, MA, USA), and one nanogram was used for library preparation by Nextera XT DNA Library Prep kit (Illumina Inc., San Diego, CA, USA) according to the manufacturer’s protocol. Deep sequencing was performed on the NextSeq 500 platform (Illumina Inc., San Diego, CA, USA) using the NextSeq 500/550 Mid Output Reagent Cartridge v2, with 300 cycles and standard 150 bp paired-end reads [12].

### 2.3. Phylogenetic Analysis

Phylogenetic analysis was performed based on the nine concatenated open reading frames (ORFs) [13,14], namely the early transcription factor/VETF enzyme large subunit (A7L), the major core protein (A10L), the RNA polymerase 132 (A24R), the messenger RNA capping enzyme the large subunit (D1R), the DNA-independent NTPase (DNA replication, D5R), the hypothetical protein (E6R9), the DNA polymerase (E9L), the RNA polymerase-associated protein (H4L), and the RNA polymerase 147 (J6R). Gene designations refer to the VACV-COP genome (GenBank accession No. M35027). The sequence dataset consisted of concatenated genome sequences of different OPXV species representative of North America and the Old World (Africa and Eurasia) that were used for alignment by Geneious version 10.1.3 and MAFFT algorithm [15]. Phylogenetic analysis was performed with Bayesian inference by using four chains run for >1 million generations [16,17]. Posterior output of the tree was derived from Bayesian inference using four chains run for >1 million generations, a general time-reversible model, a proportion of invariable sites, a gamma distribution of rate variation across sites, and a subsampling frequency of 1000. ModelTest software (http://evomics.org/resources/software/molecular-evolution-software/modeltest/) was used to identify the most appropriate model of evolution for the entire dataset and for each gene individually. The identified program settings for all partitions, under the Akaike Information Criteria, included six character states (general time-reversible model), a proportion of invariable sites, and a gamma distribution of rate variation across sites.

## 3. Results

### 3.1. Sequencing Revealed High Nucleotide Sequence Identity to Vaccinia Virus

The sequencing run of Ducapox vaccine produced a total number of 1,202,012 reads. Quality controls and trimming carried out by FASTQC (v0.11.5) and trimmomatic-0.36 [18,19], maintained 969,312 reads with an average length of 91.7 nt and mean quality of 30.73. Host depletion was performed by Bowtie 2 (v2.1.0) [20], and the remaining 74,876 reads (mean length 86.33 and mean quality 30.45) were used for *de novo* assembly by SPAdes software v. 3.11.1 [21,22] that produced a unique contig of 152,596 nt. BLASTn analysis was performed to identify the closest publicly available sequence in the GenBank database. The best match (99.99%) was with the *Vaccinia virus* strain Acambis 3000 Modified Virus Ankara (accession No. AY603355), and this sequence was used to perform a reference-based assembly by Bowtie 2 (v2.1.0) [20]. This task produced a unique consensus sequence of 166,722 nt with horizontal coverage of 96%, as two gaps of 5195 nt and 890 nt were present. Deep mean coverage was 26X. The consensus sequence was annotated using Prokka v1.12 [23] and deposited with the GenBank database (MT648498). Nt sequence identity with the five CMLV whole genome sequences available on GenBank ranges from 97.54% to 97.56%.

### 3.2. Ducapox Vaccine Clusters with Vaccinia Virus Strains

The nine ORFs (A7L, A10L, A24R, D1R, D5R, E6R, E9L, H4L, and J6R) of the Ducapox vaccine sequence were concatenated and aligned with homologous sequences of selected OPXV strains. Phylogenetic analysis was performed using Bayesian inference. Posterior probability percentages were consistently high (>95%) for all clades on phylograms, which supported inferred phylogenetic relationships.

**Figure 1:**
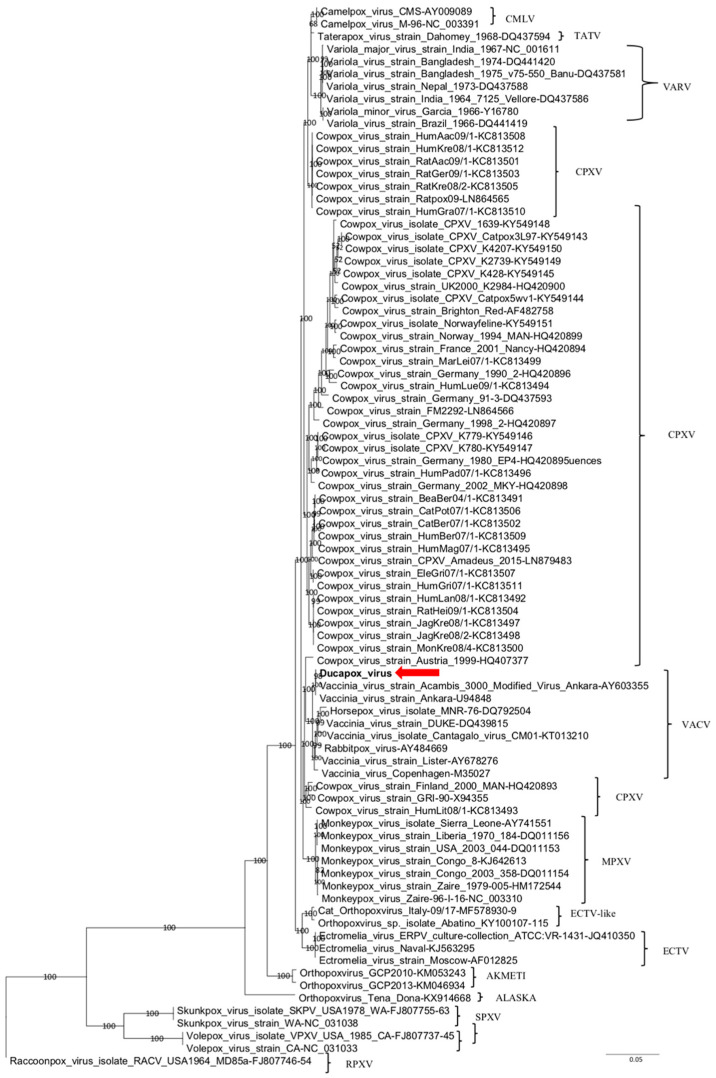
Phylogenetic relationship of extant OPXVs with Ducapox vaccine. Phylogenetic tree shows concatenated alignment of nine coding gene (A7L, A10L, A24R, D1R, D5R, E6R, E9L, H4L, and J6R) sequences of *Orthopoxvirus*. Gene designations refer to the VACV-COP genome (GenBank accession No. M35027). Posterior probability values >0.95 are indicated on the tree nodes. The red arrow indicates the Ducapox vaccine (this study). *Raccoonpox virus* strain MD85A was used as an outgroup. Scale bar indicates nucleotide substitutions per site. CMLV, *Camelpox virus*; CPXV, *Cowpox virus*; ECTV, *Ectromelia virus*; MPXV, *Monkeypox virus*; RPXV, *Raccoonpox virus*; SPXV, *Skunkpox virus*; TATV, *Taterapox virus*; VACV, *Vaccinia virus*; VARV, *Variola virus*; VPXV, *Volepox virus*.

In the phylogenetic tree (Figure 1), Ducapox vaccine was distantly related to CMLV strains; conversely, it showed a closer relatedness with VACV strains, albeit forming a separate subcluster with *Vaccinia virus* strain Acambis 3000 Modified Virus Ankara (MVA, AY603355) and *Vaccinia virus* strain Ankara (U94848) strains. Nt identity of Ducapox vaccine strain with different VACV strains ranged between 98.2 and 99.9%.

## 4. Discussion

In this study, the genome composition of Ducapox vaccine, a commercial CMLV vaccine produced by the Central Veterinary Research Laboratory (CVRL, Dubai, UAE) starting from the CMLV CaPV298-2 field strain, was investigated by NGS. The vaccine sequence content revealed the highest nt identity with an MVA strain (AY603355) of VACV and the absence of CMLV-unique regions including ORFs CMLV185, CMLV186, and CMLV187 [4]. Interestingly, nt sequences closely related to VACV strains were also revealed by Yousif and colleagues while investigating the genome composition of an additional commercially available, live-attenuated CMLV vaccine (Veterinary Vaccine Production Center (V.V.P.C.) Modified live virus vaccine for camelpox) based on the Jouf-78 field strain [8]. The only reasonable scenario explaining this bizarre situation is that contamination events had occurred independently in two different vaccines batches. Indeed, considering how viruses were handled in different laboratories around the world a couple of decades ago, such a mix-up may have happened. During those days, there were neither SOPs nor stringent disinfection protocols implemented when working with viruses. Contamination was likely when working with different viruses or strains of a particular virus at the same time, and thus exchange of viral strains was very common and not restricted in the way it is today. Newer molecular methods, such NGS, uncovered these “misguided” strains all over the world, including in unlikely hosts. Unfortunately, the original CMLV CaPV298-2 sequence is currently unknown, and evidenced-based conclusions cannot be drawn. In this regard, it is important to highlight the fact that the CaPV298-2 strain showed, after 106 passages into Vero cells, a similar deletion to that observed in MVA that originated from Chorioallantois *Vaccinia virus* Ankara (CVA) after continuous serial passages in chicken fibroblast tissue culture [24]. According to this perspective, it is tempting to speculate that contamination with MVA had occurred before the analysis of the 106th passage of the CaPV298-2 strain. This study certainly has one pitfall. Indeed, two deletions of 5195 and 890 nt with respect to the MVA strain showing the highest nt identity along the whole genome were present. The first is located in a portion of the genome encompassing the ITRs and the left terminal region, the second in the central conserved region. To date, we do not know if these deletions are “real” deletions or merely the evidence of a lack of sequence reads in those portions of the genome. Accordingly, further analyses are currently ongoing. Overall, despite what has been declared in the label, genome sequences of MVA, and not CMLV, were detected in a commercially available CMLV vaccine. However, it is also important to underline that, because all OPXVs are antigenically related, immunization with nearly any OPXV can protect against challenge with other OPXVs [25]. In fact, in the field, this Ducapox vaccine, containing MVA in place of CMLV, has been proven to be capable of protecting animals against CMLV field infection [26,27].
